# Patient-Derived Organoid Facilitating Personalized Medicine in Gastrointestinal Stromal Tumor With Liver Metastasis: A Case Report

**DOI:** 10.3389/fonc.2022.920762

**Published:** 2022-08-02

**Authors:** Ying Cao, Xi Zhang, Qianyun Chen, Xi Rao, Enming Qiu, Gang Wu, Yu Lin, Ziqi Zeng, Bin Zheng, Zhou Li, Zhai Cai, Huaiming Wang, Shuai Han

**Affiliations:** ^1^ The Second School of Clinical Medicine, Southern Medical University, Guangzhou, China; ^2^ Department of Gastrointestinal Surgery, General Surgery Center, Zhujiang Hospital, The Second Affiliated Hospital of Southern Medical University, Guangzhou, China; ^3^ Department of Oncology, Zhujiang Hospital, The Second Affiliated Hospital of Southern Medical University, Guangzhou, China; ^4^ Department of Pathology, Zhujiang Hospital, The Second Affiliated Hospital of Southern Medical University, Guangzhou, China; ^5^ Guangdong Research Center of Organoid Engineering and Technology, Accurate International Biotechnology Company, Guangzhou, China; ^6^ Department of Colorectal Surgery, The Sixth Affiliated Hospital of Sun Yat-sen University, Guangzhou, China

**Keywords:** gastrointestinal stromal tumor, patient-derived organoid, KIT exon 11 mutations, p.V560E, personalized medicine

## Abstract

The gastrointestinal stromal tumors (GIST) are a rare gastrointestinal tract malignancy. The two primary mutation sites are found in KIT and platelet-derived growth factor receptor-α (PDGFR-α) genes. The current study reports on a point mutation within the exon 11 of KIT, named KIT p.V560E. Patient-derived organoids (PDOs) are potential 3D *in vitro* models of tissues that can be used to identify sensitivity toward specific targets in patients with tumors and allow for personalized medicine when drugs specific for newly identified genetic locus mutations are not yet available. This study describes a 68-year-old patient who complained of diffused abdominal pain and intermittent melena lasting more than 10 days. He has no other gastrointestinal abnormalities, prior abdominal surgery, or related family history. Surgery was conducted first to remove the lesions and ascertain the disease through histology and immunohistochemical stains of the mass. Immunohistochemistry revealed that the tumor was positive for CD117 and Dog-1. Based on the above findings, he was diagnosed with GISTs. Gene detection analysis and organoid culture were then performed to verify clinical decisions. KIT p.V560E and the reduced number of RB1 copies were identified as two obvious mutations, so the patient was administrated first-line treatment of imatinib 400 mg/d. However, progressive disease prompted us to switch to sunitinib, and his condition gradually improved. Meanwhile, organoid culture showed sensitivity to sunitinib and tolerance to imatinib with half-maximal inhibitory concentration (IC50) values of 0.89 and >20, respectively. In summary, to the best of our knowledge, this is the first time that the established organoid culture indicated that the GISTs organoid could identify the sensitivity to target therapies and facilitate individual-based treatment.

## Introduction

As the most common mesenchymal gastrointestinal tumors, gastrointestinal stromal tumors (GISTs) account for 0.1%–3% of all gastrointestinal tract malignancy (1). GISTs are considered to originate from the interstitial cells of Cajal (ICC), the pacemaker for the peristaltic movement of the gastrointestinal tract (2, 3). These tumors are primarily the result of KIT mutations and/or platelet-derived growth factor receptor-α (PDGFR-α) mutations which activate downstream signaling and cytogenetic changes that promote tumor occurrence and progression (4). CD117 and CD34 are expressed in approximately 95% and 80% of GISTs, respectively (5) and later discovered on gastrointestinal stromal tumor 1 (Dog-1), also suggested to be a positive diagnostic marker in pathological immunohistochemistry (6). Both immunohistochemical panel (CD117/Dog-1) and molecular analysis (KIT/PDGFR-α), the gold standard, make it possible to accurately diagnose GISTs (7). The stomach (51%), the small intestine (36%), and the colon (7%) are the most common pathological entities of GISTs (8); additionally, they usually metastasize inside the abdominal cavity like the liver (50%–60%) and peritoneum (20%–43%) (9). Patients with GISTs exhibit symptoms like gastrointestinal bleeding (hematemesis, anemia, and azotemia), tiredness, abdominal pain, or intestinal obstruction (2). Current ESMO-EURACAN-GENTURIS Clinical Practice Guidelines have reached a consensus on the management of GISTs: surgical/endoscopic resection is the standard approach to tumors ≥2 cm in size, and active surveillance is suggested when the evidence for diagnosis is inadequate. Imatinib is the standard treatment for patients whose stromal tumors have progressed locally, metastasized, or are inoperative. It is also recommended for patients who well tolerated imatinib and with all the lesions removed postoperation (10). While patients with the PDGFR-α exon 18 D842V-mutation are not as sensitive to imatinib, they are significantly more responsive to this drug than to avapritinib (11). When patients are intolerant to imatinib or having advanced disease, sunitinib as the standard second-line therapy (50 mg/d 4 weeks on/2 weeks off) was approved by the Food and Drug Administration (FDA) (12). Additionally, patients with c-KIT exon 9 mutations may gain more benefits from sunitinib than imatinib treatment (13).

The novel *in vitro* 3D culture technologies, patient-derived organoids (PDOs), offer us more opportunities to study human cancer models physiologically. Even with the increased development of targeted regimens and immunotherapies for cancer, relief and recovery from tumors remain a significant challenge. Current animal models cannot perfectly mirror human tumors, simulate progression, or identify genetic heterogeneity, making it difficult to translate findings into clinical practice (14). Therefore, patient-derived cancer organoids are being prioritized for use in guiding personalized medicine. Thus far, no precedents have reported the utilization of PDOs to test the sensitivity toward KIT‐targeted inhibitors in patients with GISTs. The current case report describes a GIST patient with liver metastasis and identifies a role for PDO in optimizing treatment and informing clinical decision-making.

## Case Presentation

**Figure 1:**
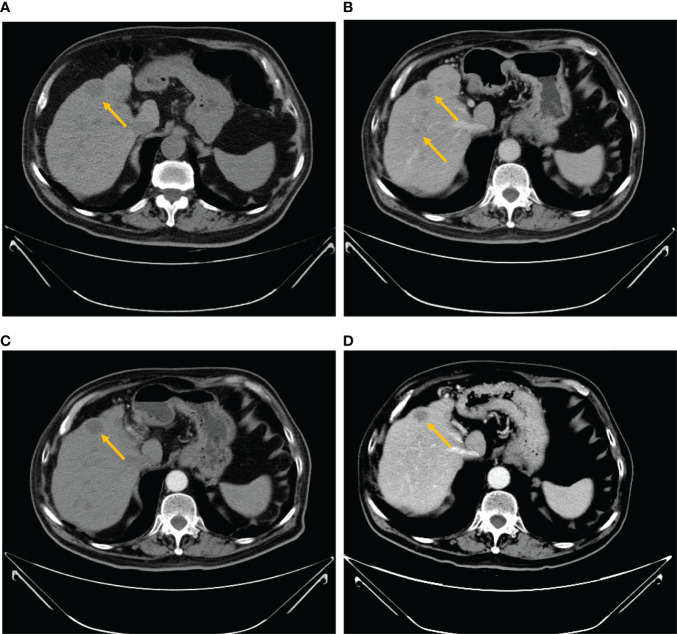
Abdominal computed tomography scan. **(A)** The baseline CT scan performed on August 26, 2021 showed multiple large hypodense lesions in the liver. Contrast-enhanced CT showed ring-shaped enhanced nodules with the maximum diameter of 17 mm. **(B)** After 2 months of treatment with imatinib, a CT scan was performed on October 25, 2021, a relapse of disease (growth of the longest lesion’s diameter from 17 to 25 mm) of the hepatic lesion and multiple hepatic metastases with slight reinforcement was observed. **(C)** The venous phase of the CT scan performed on November 25, 2021 demonstrated decreased hypodense lesions (growth of the longest lesion’s diameter from 25 to 23 mm) with peripheral rim enhancement. **(D)** After two cycles of sunitinib, a CT scan was performed on January 19, 2022 and revealed a smaller, irregular hypodense intrahepatic metastatic mass.

A 68-year-old man with a diagnosis of primary hypertension presented to the general surgery department on August 24, 2021 for diffused abdominal pain and intermittent melena lasting more than ten days. The man denied other gastrointestinal abnormalities, prior abdominal surgery, or related family history. Abdominal tenderness, especially in the epigastric, tenderness without rebound tenderness or Murphy’s sign was observed in the physical examination on admission. His blood test results revealed that he was anemic, with red blood cell (RBC), hemoglobin (Hb), hematocrit value (Hct), and mean corpuscular hemoglobin concentration (MCHC) of 3.3×10^12^/L [normal range (4.3–5.8) × 10^12^/L], 94 g/L (normal range, 130–175 g/L), 0.3 L/L (normal range, 0.4–0.5 L/L), and 312 g/L (normal range, 316–354 g/L), respectively. Liver and kidney function and electrolyte levels showed results within the normal range. In addition, no abnormality was observed in his serum levels of carbohydrate antigen (CA) 199 was 9 KU/L (normal range, <34 KU/L) and carcinoembryonic antigen (CEA) was 1.1 ng/ml (normal range, ≤5 ng/ml). A computed tomography (CT) scan indicated small liver lesions with multiple hypodense nodules about 30–33 Hu value, and in contrast-enhanced CT observed ring-shaped enhanced nodules with a maximum diameter of 17 mm (**Figure 1A**). The CT report considered liver cirrhosis and possible liver metastases that required confirmation based on clinical symptoms and other examination results. The capsule endoscopy found an ulcerated bulge covered with yellow-white digesta and bloodstains (**Supplementary Figures 1A–C**). Narrowing of the intestinal lumen required a slow descending of capsule endoscopy, delaying and terminating intestinal inspection. Additionally, abundant fresh blood was visible beside the bulge. Abdominal plain film examination revealed no expansion of the enteric cavity, gas-fluid, or subphrenic air.

**Figure 2:**
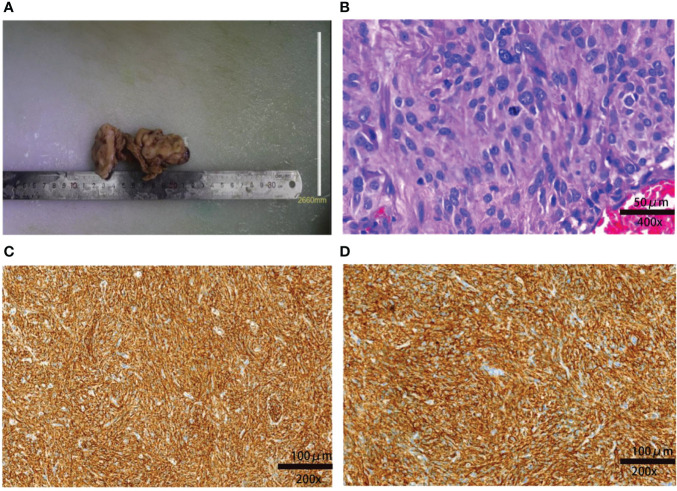
Macroscopic and microscopic findings of the resected tumor. **(A)** The resected specimen of the small intestine measured 10 × 5 × 2 cm. **(B)** Postoperative pathology indicated that the tumor was a high-risk GIST: the lesion had significant nuclear pleomorphism with mitotic >10/50 HPF (hematoxylin and eosin staining). The spindle or ovoid cells are deeply stained with coarse chromatin and obvious atypia (×400). **(C)** Immunochemical staining showing that the tissue was CD117+ (×200). **(D)** Immunochemical staining showing that the tissue Dog-1+ (×200).

Surgery of resecting intestinal and liver metastases was conducted on September 1, 2021 to conduct a hemostasia operation and ascertain the disease through pathological and immunohistochemical stains of the small intestine and liver masses: GIST (small intestine, liver), high risk, and mitotic >10/50 HPF. Immunohistochemistry results were CD117 (+), Dog-1 (+), smooth muscle actin (+), Vim (+), CK (−), CD34 (−), and SOX-10 (−) (**Figures 2A–D**). Meanwhile, gene detection and organoid culture were performed verify the clinical diagnosis. Targeted genetic tests using next-generation sequencing of the resected tumors from the small intestine and liver were performed to clarify somatic gene mutation: we observed two significant gene mutations, KIT p.V560E and the reduced number of RB1 copies. KIT p.V560E indicated that the valine in the 560 codon of the KIT gene was mutated to glutamate, and it was within the exon 11 of KIT, and KIT mutation accounts for 60% of GISTs (**Supplementary Figures 2A, B**) (15). We also analyzed the sensitivity and applicability to immunotherapy: microsatellite stable; microsatellite instability where the tumor mutational burden (TMB) was rated medium of 2.23 Muts/Mb (mutational load per million bases), lower than 57% of patients with GISTs (small intestine); and no mismatch-repair gene deficiency detected (**Supplementary Figures 2C, D**).

**Figure 3:**
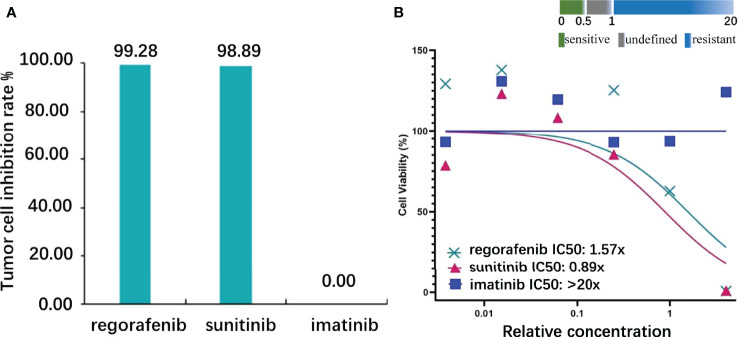
Target drug susceptibilities. **(A)** The inhibition rate of the highest concentration: regorafenib, sunitinib, and imatinib were 99.28, 98.89, and 0.00, respectively (the control cells received no treatment, and the cell viability was 100%). **(B)** Half-maximal inhibitory concentration (IC50): regorafenib, sunitinib, and imatinib were 1.57, 0.89, and >20, respectively (definition: sensitive, IC50<0.5; undefined, 0.5<IC50<1; resistant, IC50>1).

**Figure 4:**
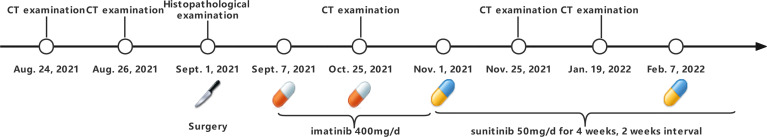
Timeline of the diagnostic and therapeutic process.

Subsequently, we also established the organoid model with small intestinal during surgical resection (**Supplementary Figures 3A–C**) to assess the drug response to currently widely used KIT-targeted drugs (16). The liver organoid was also cocultured but the cell viability was inferior to the small intestinal organoid. The genetic testing results obtained from the small intestine and liver had the same gene mutation sites, so the small intestine organoid could predict treatment response that corresponded with the patient. Briefly, the patient tumor tissue was minced and digested into small cell clusters (**Supplementary Figure 3A**) and passed through a 70-μm filter. The cell suspension was then mixed with the Matrigel matrix (Corning Inc, Corning, NY), transferred to a culture plate, and incubated at 37°C and 5% CO_2_ cell culture incubator for 30 min. On complete gelation, the culture medium was added and cultured until enough PDOs were formed (**Supplementary Figures 3B, C**). Both the hematoxylin–eosin and immunochemical staining demonstrated that cultured PDOs retained key phenotypic characteristics of the parent GISTs like nuclear pleomorphism, mitotic rate, and immunoreactive profiles (**Supplementary Figures 3D, E**). The maximal tumor inhibition was 98.89% for sunitinib and 99.28% inhibition for regorafenib. The drug sensitivity of GIST-PDO against widely used target drugs including imatinib, sunitinib, and regorafenib was examined. To compare the drug sensitivities of the tested drugs, the relative half-maximal inhibitory concentration (IC50) of each drug was determined using the “Accurate drug sensitivity cut-off database,”. The IC50 of each drug can be divided into sensitive (0–0.5), undefined (0.5–1), and resistant (>1) groups. The concentration–response curves manifested that PDOs were resistant to both imatinib (IC50: >20×) and regorafenib (IC50: 1.57×), and sensitive to sunitinib (IC50: 0.89×) (**Figure 3**). Although regorafenib has a cytotoxic effect on neoplastic cells, it was not recommended to the patient, as its IC50 surpassed 1×, and sunitinib was ranked the optimal regimen according to the PDOs results. Notwithstanding, we administrated imatinib 400 mg daily to the patient a week postoperation, the standard first-line treatment FDA-approved treatment. Approximately 2 months later, the patient complained of epigastric pain and CT indicated more enlarged nodules with a maximum diameter of 25 mm, increased parietal thickness, and increasing nodules (**Figure 1B**). Thus, the therapy was switched to sunitinib (continuous 50 mg/d for 4 weeks with a 2-week interval) on November 1, 2021. CT demonstrated a well-defined, shrunk homogeneous soft-tissue mass on November 25, 2021 (**Figure 1C**). By Jan 19, 2022, CT scans showed partial lesion absorption (**Figure 1D**). The patient expressed abdominal pain relief demonstrating that he had experienced a partial response (PR). During the whole diagnosis and treatment periods, his CA199 and CEA remained normal. The diagnosis and treatment strategy timeline schematic is presented in **Figure 4**.

## Discussion

The first application of organoid culture in 2009 (17) opened a new era for cancer research by allowing researchers and clinicians to observe the tumors’ biological features, discover novel biomarkers, and improve personalized treatments. Organoids derived from surgical procedures or tumor biopsies can inform clinical decision-making by providing a mechanism for reliably testing drug sensitivity and IC50 value (18). Meanwhile, large cohorts and randomized controlled trials can then be used to validate the results of organoids or, paralleled with genetic testing, to implement individualized cancer therapy.

Here, we report a case of a patient of GIST with liver metastasis whose response to treatment matched the intestine organoid culture results. Surgical resection (reaching the greatest extent possible) and segmental liver resection with laparoscopic surgery were recommended as the first therapeutic option in order to eliminate the possible life-threatening symptom of melena, determine the accuracy of the diagnosis using histological and immunohistochemical stains of the tumor, activate cancer cells’ sensitivity to adjuvant therapy as a result of the decreased tumor load, and preserve tumor tissue for organoid development to assist clinical decision-making (19). Regular postoperation monitoring and supplementary target therapy are essential for a better prognosis. A retrospective study reported that resection of liver metastases in GIST patients combined with imatinib may lead to improved prognosis with 1- and 3-year progression-free survival of 93% and 67% respectively (20). The case reported here was not appropriate for immunotherapy: because he was microsatellite stable (MSS), had a medium TMB, and was no mismatch-repair gene deficient. A high TMB may be associated with a positive response to immunotherapy, but the cutoff point is dependent on where cancer originated (21, 22). The TMB of our patient was lower than 57% of small intestinal GIST patients. Molecular genotyping results demonstrated that mismatch-repair deficient or microsatellite instability-high colorectal cancer have adequate immune activation required to respond with immunotherapeutic agents (23, 24). Therefore, imatinib 400 mg/d was administrated to the patient as the standard first-line therapy, however, GIST progression was observed a month later. Research indicates that patients with KIT exon 11 mutation appeared to benefit less than whose with the KIT exon 9 mutation when imatinib is increased to 800 mg/d to halt disease progression (25). As a result, the case reported here was switched to sunitinib 50 mg/d for 4 weeks followed by a 2-week rest (26). The patient’s right epigastrium pain was relieved after being administrated with sunitinib, and CT scans revealed the presence of homogeneous shrunk lesions.

In this case report, we sought to explore the reasons for liver metastases’ recurrence and disease progression. On the one hand, several studies have confirmed that KIT-associated tumors progression when combined with additional sporadic mutations (27, 28), such as the decreased RB1 copies seen in this case. This could potentially incur GISTs’ metastasis in the liver. On the other hand, it was expected that imatinib treatment would improve recurrence-free and overall survival of this high-risk patient (29). Instead, the KIT p.V560E appeared to incur resistance to imatinib, a finding not reported previously. Generally, it is acknowledged that KIT exon 9 mutations or GIST without PDGFR-α or KIT mutations are more likely to acquire resistance than KIT exon 11 mutations, accounting to 10% of advanced GISTs patients (30, 31). Our patient’s gene detection reported KIT p.V560E, whose valine in the 560 codon of the KIT exon 11 gene was mutated to glutamate. A previous study found that motesanib could inhibit autophosphorylation of KIT mutants V560D more potently than imatinib in transfected Ba/F3 cells, with IC50 values of 3 and 7 nM, respectively (32). In our case, we consider that sunitinib could exhibit superior efficacy than imatinib, with IC50 values of 0.89 and >20, respectively. The possible mechanical explanation could be that mutated glutamate changed the juxtamembraneous domain of KIT, small sunitinib may bind to the ATP-binding pocket of the KIT protein, and this gatekeeper mutation hindered the incorporation of large imatinib (33).

Of note, the postoperative efficacy of the chosen drugs was consistent with the results obtained from suggesting that tumor organoids could inform treatment decisions because they could retain the original cancer gene mutation. Other examples of successful organoid use are evident in the literature. A recent case of oligometastatic colorectal cancer, for example, underwent surgical resection and followed systemic FOLFOX treatment regimen. However, the prognosis was not as expected; the patient relapsed and a clinical decision was made to switch to 5-fluorouracil and SN-38 treatment based on pharmacologic organoid screening. The case exhibited promising tumor shrinkage and experienced a partial recovery, and this case could inform us of the organoids’ role in drug sensitivity testing, supporting personalized clinical choice (18). Vlachogiannis et al. used a living biobank of patient-derived organoids collected from pretreated metastatic colorectal and gastroesophageal cancer tissues to identify PDOs’ credibility to predict clinical efficacy (34). The results were courageous because PDOs could recapitulate original tumor mutations and match drug monitoring susceptibility of the patient.

Current treatments focus on precise and individualized medicine for different genome and transcriptome landscapes, lifestyles, and progressive disease courses. The novel prominent choice, gene-targeting therapy, emerged to be powerful; however, not all patients could gain the expected effect of the recommended target drugs (35), as our patient reported above. Exact explanations from theoretical mechanisms remain challenging. In general, patient‐derived organoids could potentially compensate for this gap through their ability to retain the original mutation of the patient tumor and recapitulate drug responses. Organoid culture results could be a promising supplement or alternative to gene detection; moreover, it can be used to elucidate possible genetic alterations linked to drug resistance. For example, if a patient acquires secondary resistance to sunitinib and the combination of sunitinib, its downstream signaling, mammalian target of rapamycin (mTOR) may be a promising strategy (36). Notwithstanding credible efficacy data, we could utilize the organoid culture technology to provide preliminary validation.

While PDOs encountered dramatic progression in clinical therapy over the past decade, their intrinsic property limited their advance. Because the tissues or tumors are acquired from the individual patient, heterogeneity can attribute to diverse treatment options that make consensus a challenge. Besides, not all tumors could adapt to the external environment or retain their original mutations in vitro (37). Growth rates varied among different tumor tissues and some tumors, such as breast cancer, may take 6 months to become organoids (38), so fastened culture processes despite neoplasms‘ histological type are urgently needed. Additionally, it is critical to maintain the primary genetics of the tumor. Maintaining primary genetics is foremost. In the future, standardization of organoid culture and identification process, improvement of culture success rate, accurate drug sensitivity detection methods, and optimization of drug sensitivity related parameters still need to be ascertained; especially, large cohort clinical trials are essential to validate the patient‐tailored treatment. We believe that following additional clinical observational and interventional studies, the organoid models will inevitably be regularly used to improve the cancer therapy and patients’ quality of life.

In conclusion, this study describes the first reported use of GISTs’ organoids to identify sensitivity to target drugs and facilitate individual-based treatment. The results indicated that GISTs with KIT p.V560E may be more sensitive to sunitinib than imatinib, suggesting that sunitinib may be a preferred treatment in the treatment of GISTs with KIT p.V560E. Furthermore, our study demonstrated that GIST-PDO could represent a faithful tumor model and validate drug responses *in vivo*; it may be promising to combine current guidelines with PDO results before initiating treatment to elucidate possibly the most appropriate regimens and advance precision cancer medicine.

## Data Availability Statement

The original contributions presented in the study are included in the article/**Supplementary Material**. Further inquiries can be directed to the corresponding author.

## Ethics Statement

Written informed consent was obtained from the individual(s) for the publication of any potentially identifiable images or data included in this article.

## Author Contributions

Material preparation and data analysis were performed by XZ, EQ, and GW. The first draft of the manuscript was written by YC, QC, XR, and HW. ZL, ZC, GW, and SH treated the patient. BZ and his affiliated company conducted the organoids culture. SH is the guarantor of this work and, as such, had full access to all of the data in the study and takes responsibility for the integrity of the data and the accuracy of the data analysis. All authors contributed to the article and approved the submitted version.

## Conflict of Interest

BZ was employed by Accurate International Biotechnology Company.

The remaining authors declare that the research was conducted in the absence of any commercial or financial relationships that could be construed as a potential conflict of interest.

## Publisher’s Note

All claims expressed in this article are solely those of the authors and do not necessarily represent those of their affiliated organizations, or those of the publisher, the editors and the reviewers. Any product that may be evaluated in this article, or claim that may be made by its manufacturer, is not guaranteed or endorsed by the publisher.

## Supplementary Material

Supplementary Figure 1 Capsule endoscopy. **(A)** A swelling mass found in the upper small intestine. **(B)** Local ulceration, including abundant fresh blood, yellow-white digesta and food scraps were observed. **(C)** Narrow intestinal lumen delayed observation of the capsule endoscopy. The capsule remained in the middle part of the small intestine so the examination was not completed.

Supplementary Figure 2 Results of genetic testing. **(A)** KIT 560point mutation identified in next-generation sequence read from tumor tissue: the novel point mutation (V to E) was identified within the KIT gene. (V=Val=Valine; E=Glu=Glutamic acid) **(B)** Gene copy number changes in this patient’s sample: no mutations of clinical significance were observed. (The horizontal axis represents the location of the chromosomes. The vertical axis represents next-generation sequencing (NGS)-based copy number variant (CNV) detection for the analyzed tumor sample). **(C)** MSS: the patient’s MSI score was much lower than the cutoff. **(D)** TMB was 2.23Muts/Mb, lower than 57% small intestinal GIST patients. (TMB is defined as mutational load per million bases (Mb) in tumor within targeting coding regions. high: 0-25% medium; 26-75% low; 76%-100%).

Supplementary Figure 3 Images of organoid culture cells. **(A)** The patient tumor tissue was minced and digested into small cell clusters. **(B, C)** Microscopic image of culture cells showing an intermediate proliferation rate and activity. There were 2000 tumor organoids with the average diameter of 41.11μm. **(D)** Hematoxylin and eosin staining of culture cells. (× 400). **(E)** Immunochemical staining showing that Dog-1 was positive. (× 400).

## Abbreviations

GIST, gastrointestinal stromal tumor; PDGFR-α, platelet-derived growth factor receptor-α; PDOs, patient-derived organoids; ICC, interstitial cells of Cajal; Dog-1, discovered on gastrointestinal stromal tumor 1; RBC, red blood cell; Hb, hemoglobin; Hct, hematocrit value; MCHC, mean corpuscular hemoglobin concentration; CA, carbohydrate antigen; CEA, carcinoembryonic antigen; TMB, tumor mutational burden; Muts/Mb, mutational loads per million bases; IC, inhibitory concentration; MSS, microsatellite stable; mTOR, mammalian target of rapamycin.
