# Supplementary figures and images for: Patient-Derived Organoid Facilitating Personalized Medicine in Gastrointestinal Stromal Tumor With Liver Metastasis: A Case Report
Source: Front Oncol. 2022 Aug 2;12:920762. doi: 10.3389/fonc.2022.920762 (PMC9378866; doi:10.3389/fonc.2022.920762)

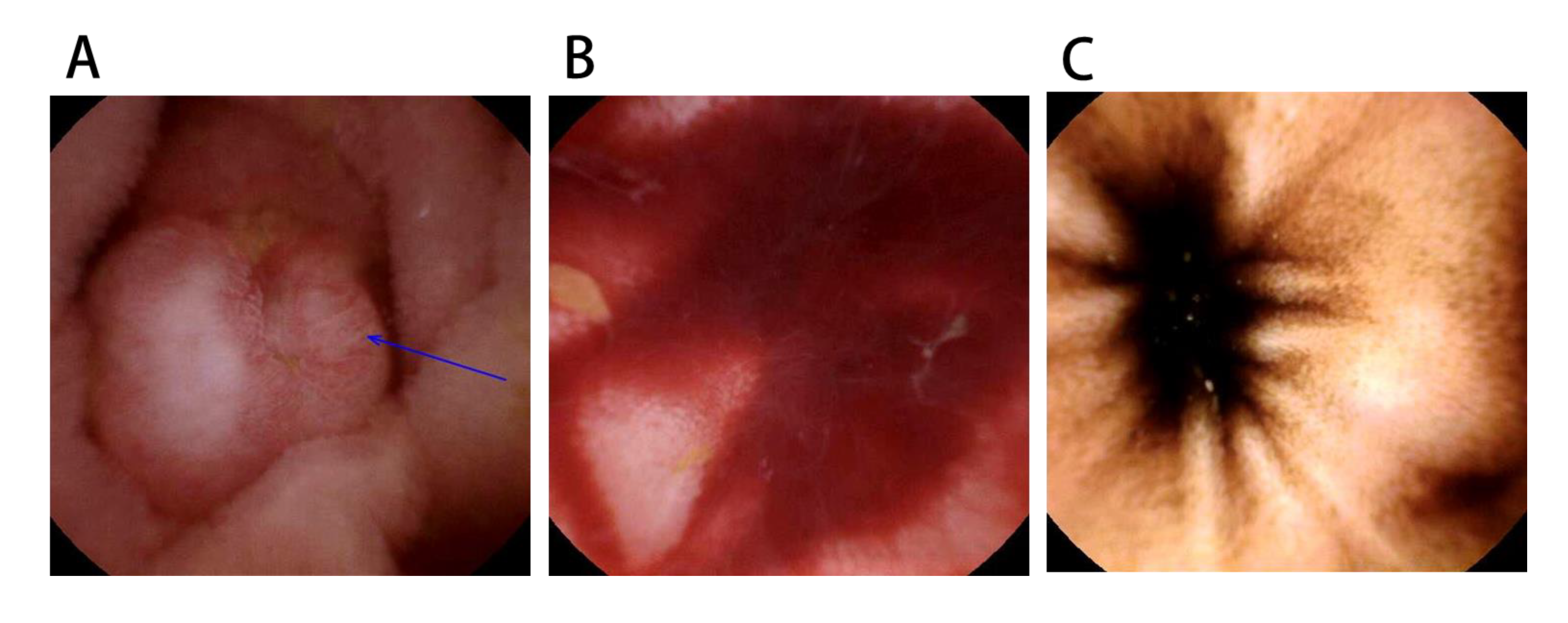

Supplement: Supplementary Figure 1 — Capsule endoscopy. (A) A swelling mass found in the upper small intestine. (B) Local ulceration, including abundant fresh blood, yellow-white digesta and food scraps were observed. (C) Narrow intestinal lumen delayed observation of the capsule endoscopy. The capsule remained in the middle part of the small intestine so the examination was not completed. [file Image_1.tif]

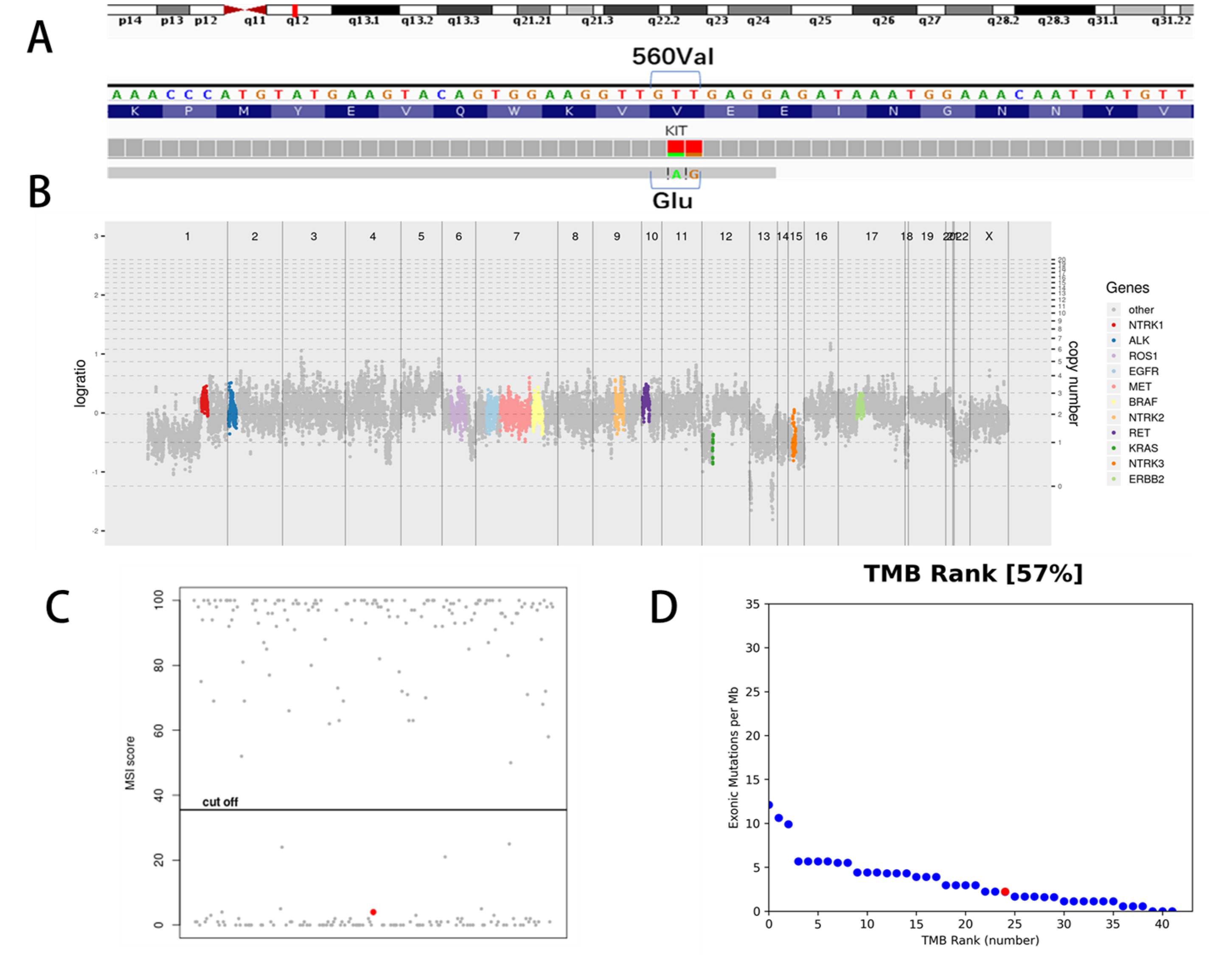

Supplement: Supplementary Figure 2 — Results of genetic testing. (A) KIT 560point mutation identified in next-generation sequence read from tumor tissue: the novel point mutation (V to E) was identified within the KIT gene. (V=Val=Valine; E=Glu=Glutamic acid) (B) Gene copy number changes in this patient’s sample: no mutations of clinical significance were observed. (The horizontal axis represents the location of the chromosomes. The vertical axis represents next-generation sequencing (NGS)-based copy number variant (CNV) detection for the analyzed tumor sample). (C) MSS: the patient’s MSI score was much lower than the cutoff. (D) TMB was 2.23Muts/Mb, lower than 57% small intestinal GIST patients. (TMB is defined as mutational load per million bases (Mb) in tumor within targeting coding regions. high: 0-25% medium; 26-75% low; 76%-100%). [file Image_2.tif]
